# An Interchangeability Test Method for Developing IPG Impedance Equivalency Evidence for Cardiac Implantable Electronic Device Under MRI

**DOI:** 10.1002/mrm.70389

**Published:** 2026-04-26

**Authors:** Hongbae Jeong, Andrew Smith, Ananda Kumar

**Affiliations:** ^1^ Office of Science and Engineering Laboratories Center for Devices and Radiological Health, U.S. Food and Drug Administration Silver Spring Maryland USA; ^2^ Office of Cardiovascular Devices Center for Devices and Radiological Health, U.S. Food and Drug Administration Silver Spring Maryland USA

**Keywords:** active implantable devices, CIED, mixed‐manufacturer system, MR safety, pulse generator, RF‐induced heating

## Abstract

**Purpose:**

Patients with cardiac implantable electronic devices (CIEDs) may receive replacement device components over time, resulting in mixed‐manufacturer systems that pose unknown, potentially higher risk under MRI exposure. It is not clear how to efficiently evaluate whether adding a non‐original implantable pulse generator (IPG) changes the system's radiofrequency (RF)–induced heating response. This study aimed to develop a least‐burdensome RF safety evaluation method for MR Conditional labeling of CIEDs. Critical parameters were characterized and demonstrated as an IPG equivalency test method that supports leveraging existing single‐manufacturer MR‐Conditional labeling for mixed‐manufacturer systems.

**Methods:**

The transfer function model of the pacing lead was measured using the original system (Lead_A_ + IPG_A_) or a third‐party IPGs (Lead_A_ + IPG_B_ or IPG_C_). Deposited power near the lead tip was compared for 50 Ω and open‐end conditions, and lead or IPG impedances were measured at 64 and 128 MHz.

**Results:**

Lead tip heating was not significantly different between original and third‐party IPGs configurations (*p* < 0.001). RF‐induced heating responses were outside of measurement uncertainty when the lead was connected with a 50 Ω resistor and some open‐end conditions. The results showed that small changes in IPG impedance (< 2.5 Ω) to the lead‐IPG system impedance (> 80.2 Ω) may not significantly alter RF‐induced heating, remaining within measurement uncertainties.

**Conclusion:**

A simplified IPG impedance equivalency test has been developed to facilitate MRI access for patients with CIEDs that undergo IPG interchange. This test aids assessment of whether replacing an original IPG model with a different model may significantly increase RF‐induced heating compared to the original system.

## 1 Introduction

More than 10 million patients undergo Magnetic Resonance Imaging (MRI) in the United States each year [1]. Beyond risks from magnetically induced force and torque due to strong static magnetic fields, MRI scanners produce time‐varying electromagnetic (EM) fields; their interaction with the human body is complex. This is especially concerning for patients with active implantable medical devices (AIMDs), where RF fields can couple to the metallic leads [2] (the “antenna effect”), leading to localized heating that could potentially cause severe thermal damage to tissues [3, 4].

Due to such risks, cardiac implantable electronic devices (CIEDs) are typically tested to support MR Conditional labeling that ensures patient safety under MRI scans. The International Standards Organization (ISO) Technical Specification (TS) 10974 [5] defines methods to assess the safety of AIMDs in the MR environment. CIED systems are often composed of implantable pulse generators (IPGs) and elongated insulated leads. Over time, a patient's physician may advise replacing the IPG in a CIED system due to battery depletion, device malfunction [6], or changes in patient health necessitating upgrades to the system. While IPG replacement is a straightforward procedure, indwelling connected leads can be difficult to replace due to biological adhesion generated by the host tissue. The same vendor and model of IPG may not always be available as an option, leading to replacement with a third‐party IPG model to deliver the intended therapy. However, whether this introduces additional risk is difficult to assess without additional testing [2]. Per single‐center experience reported by König et al. [6], nearly 30% of patients already had mixed‐manufacturer CIED systems among 127 patients who underwent MRI scans between 2013 and 2020. It is unclear how to assess the MR Conditional labeling for mixed‐manufacturer systems; a case‐by‐case risk–benefit analysis may be performed by a multidisciplinary team that may be available in the hospital. Otherwise, the mixed‐manufacturer system could be a reason for the exclusion in MRI scans. For this reason, the Heart Rhythm Society has called for action from manufacturers, academia, and regulators to investigate the safety of mixed‐brand CIEDs in MRI and identify cross‐compatible systems, to advocate for patient access to MRI [7].

Here, we developed an IPG interchangeability test method using the transfer function (TF) model defined in ISO/TS 10974 [5], and an additional test method to assist that the RF‐induced heating characteristics remain equivalent when replacing the original IPG with a third‐party IPG. The original CIED system is tested for the TF model along the lead trajectory, and the resulting heating at the lead electrode is assessed under 20 different exposure scenarios at 64 and 128 MHz. The tests are then repeated using third‐party IPGs from two different manufacturers. The results are compared with two controlled conditions: the lead connected to a 50 Ω load and open‐circuit termination.

## 2 Methods

### 2.1 TF Model Measurement

**FIGURE 1:**
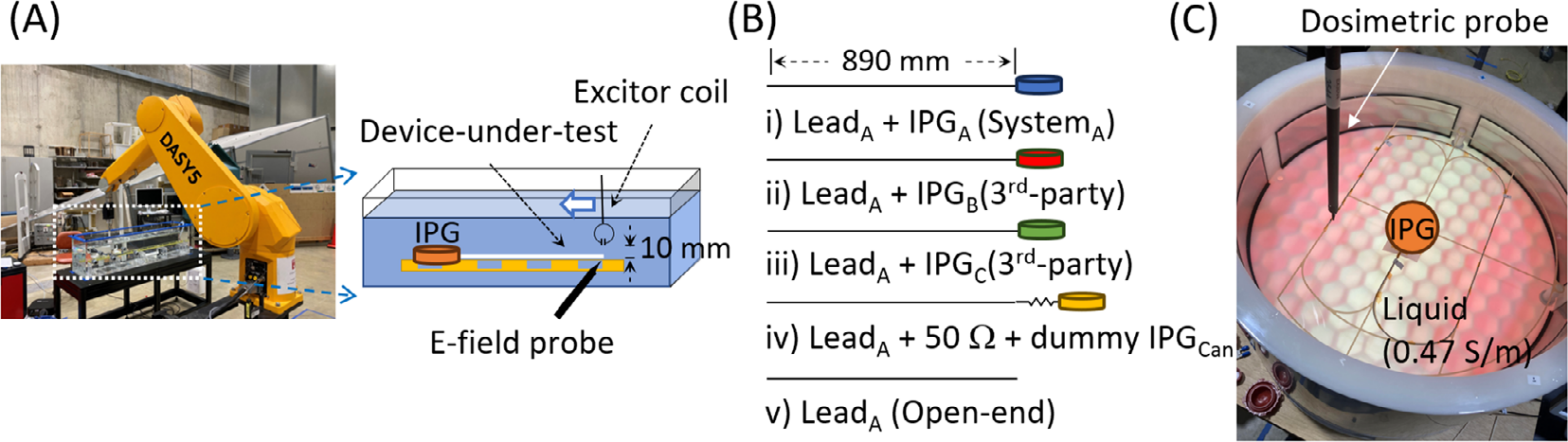
Overview of experimental setup. (A) Transfer function model measurement using a piecewise excitation method; (B) Five experimental measurement conditions for 890 mm pacing lead (Lead_A_) connected with an original and third‐party IPGs (IPG_B_, IPG_C_), a 50 Ω resistor, and remaining open‐ended; (C) Device‐under‐test positioned on the oval‐shaped racetrack inside a tabletop E‐field generator (MITS‐TT).

The piecewise excitation method [8], piX (Zurich MedTech AG; ZMT, Switzerland), was used to measure device TF models at both 64 and 128 MHz (Figure 1A). Representative CIEDs were selected from three different manufacturers (e.g., A, B, C), and IPGs were programmed in a specific mode called “MRI Mode”: the sensing system is disabled to prevent misinterpretation of cardiac signals during MRI while delivering continuous electrical stimulation at a fixed rate (asynchronous pacing). Two pacing leads (Lead A length: 890 mm, Lead C length: 550 mm) and pacemakers from three vendors were positioned on a straight FR‐4 glass reinforced epoxy racetrack, firm securing the device using Kapton tape for the TF measurement (i. Lead_A_‐IPG_A_; System_A_) and submerged in conductive tissue‐simulating liquid (TSL; *σ*: 0.47 S/m, *ε*_*r*_: 78; TLe78c0.47, ZMT). The piX excitor coil tuned at 64 and 128 MHz was attached to a robotic arm (Dosimetric Assessment System; DASY52, Schmid and Partners Engineering AG; SPEAG, Switzerland), which was swept along the device trajectory with uniform tangential E‐field exposure at every 10 mm. The pacing lead electrode response due to piX excitation along the tangential direction was measured near the lead electrode using an optical E‐field probe (E1TDSz, SPEAG). The background response was measured without the pacing lead and IPG by sweeping the piX excitor coil along the same TF model measurement trajectory, which was later used to subtract the excitor response from the TF model measurement. The measured TF model was then normalized such that the integral of TF magnitude over the pacing lead length equaled 1 to assess the TF model shape changes per IPG impedance. After completing the measurement of RF‐induced heating of SystemA, the original IPG_A_ was replaced with a third‐party IPG_B_ and IPG_C_ (i.e., ii. Lead_A_ + IPG_B_, iii. Lead_A_ + IPG_C_), and the TF model measurement was repeated under the same piX excitation conditions while maintaining the pacing lead and optical E‐field probe in the same position. Additionally, a 50 Ω resistor was connected between the Lead_A_ electrode pin and IPG enclosure surfaces (IPG_Can_) in series using copper tape on the IPG housing (iv. Lead_A_ + 50 Ω), followed by a lead open‐ended condition without IPG (v. Lead_A_; Open‐end) (Figure 1B). The experiment was repeated using System_C_ (Lead_C_ + IPG_C_).

### 2.2 RF‐Induced Power Measurement Near Pacemaker Lead

We measured the pacing lead responses under diverse test fields using a commercial tabletop E‐field generator (MITS‐TT, ZMT). The device‐under‐test (DUT) was placed on the oval‐shaped FR‐4 glass reinforced epoxy racetrack using Kapton tape prepared to guide the elongated device inside the MITS‐TT at a distance of 50 mm away from the wall in a vertically centered position. The test sample was exposed to diverse test fields [9] using a tabletop E‐field generator filled with TSL, at 64 and 128 MHz (Figure 1C). Twenty different incident E‐field exposure conditions were used (Table S1) to test RF‐induced heating near the lead tip electrode by changing the relative amplitude and phase of the two‐channel transmit sources (input power: 39 dBm) while measuring the deposited power near the lead electrode tip using a dosimetric E‐field probe (EX3DV4; SPEAG). A volume scan was performed above the lead tip (10 mm × 10 mm × 3 mm) at the exposure condition that produced the largest power near the lead electrode. Finally, the device was removed, and a background scan was conducted at each test field along a 20‐cm straight line to scale the measurement results. The measurement probe location error was removed by registering the volume scan data to the estimated −30 dB RF‐induced power near the lead electrode using a Python notebook tool called “*Touchless*,” as described in previous studies [10, 11].

The uncertainty of the TF model measurement and tabletop E‐field exposure system was estimated as reported in previous publications [10, 11, 12], where the measurement equipment uncertainty was provided by the vendor [13]. The uncertainty of the tabletop E‐field generator field exposure was assessed by running a series of simulations in which the maximum deviation in relative amplitude and phase differences during the experiment was compared to the deposited power expected at target relative amplitude and phase differences to gauge their impact on the RF‐induced power results. Total standard uncertainty (*k* = 1) was calculated by root‐sum‐square of each term of standard uncertainty following ISO/IEC Guide 98‐3:2008 [14].

### 2.3 IPG Impedance Measurement

IPG impedances between IPG_Pin_ and IPG_Can_ were measured using a Vector Network Analyzer (VNA) (P5004A; Keysight Technologies, USA) at 64 and 128 MHz. The inner conductor in the coaxial cable connected to the VNA port 1 was placed in contact with the IPG connector pin for the electrode (IPG_Pin_) while the coaxial ground was connected to the IPG enclosure surfaces (IPG_Can_). Impedance measurements were performed three times for each condition. Similarly, lead impedances between Lead_Pin_ and IPG_Can_ were measured using a VNA at 64 and 128 MHz with a customized jig that connected the signal to the lead pin end and the ground to the IPG_CAN_, while both the lead and IPG_CAN_ were submerged inside the TSL.

## 3 Results

### 3.1 TF Model Measurement

**FIGURE 2:**
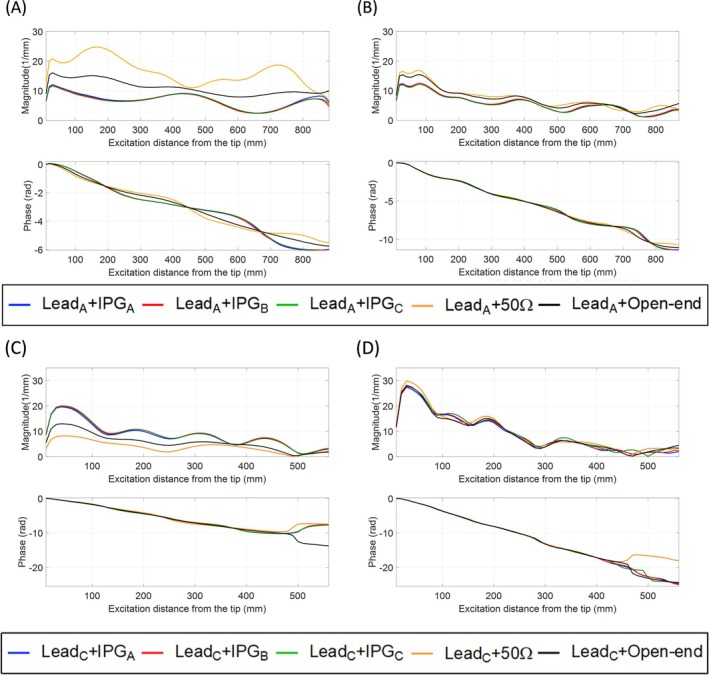
Measured transfer function model of a pacing lead. (A) Normalized transfer function model $\left(1=\int_{0}^{L}\mathrm{TF}\left(l\right)\mathrm{dl}\right){\left(\int_{0}^{L}\mathrm{TF}\left(l\right)\mathrm{dl}\right)}^{*}$ of five conditions at 64 MHz (1.5 T); and (B) at 128 MHz (3 T) using Lead A; (C) 64 MHz (1.5 T); and (D) 128 MHz (3 T) using Lead C (NRMSE and correlation coefficients were calculated using the magnitude‐only data).

The TF model measurement of an 890 mm lead (Lead_A_) took 12 min, and a 550 mm lead (Lead_C_) took 8 min, and the MITS‐TT test took less than 30 min for one device configuration. The scaled measured TF models of the Lead_A_ in five IPG connection conditions at 64 and 128 MHz are shown in Figure 2. A comparison of TF models of the pacing lead with four IPG impedance conditions is presented in terms of magnitude (1/mm) and phase (radians). The changes in the magnitude of the TF model were evaluated using the normalized root‐mean‐square error (NRMSE) and correlation. The NRMSE was 0.06 and 0.04 with a correlation (*r*) of 0.99 or higher when IPG_A_ was replaced by IPG_B_ and IPG_C_ compared to the 50 Ω or open‐end conditions, which had NRMSE values of 0.62 and 0.40, and correlations of 0.11 and 0.70, respectively, at 64 MHz. At 128 MHz, the NRMSE between the TF model of SystemA versus Lead_A_ with the IPG_B_ and IPG_C_ were 0.01 and 0.04 with a correlation of 0.99, respectively. The NRMSE increased slightly to 0.26 when Lead_A_ was connected with a 50 Ω resistor (*r* = 0.95), and to 0.20 with a correlation of 0.98 compared to the original SystemA when Lead_A_ was in the open‐end condition at 128 MHz. The RF‐induced voltage TF model results are shown in Figure S1. SystemA showed moderate correlation in voltage TF model shape when compared to the Lead_A_ connected to IPG_B_ (*r* = 0.95), or IPG_C_ (r = 0.79), a 50 Ω resistor (*r* = 0.44), and when it remained open‐ended (*r* = 0.06).

### 3.2 RF‐Induced Power Measurement Near Pacemaker Lead

**FIGURE 3:**
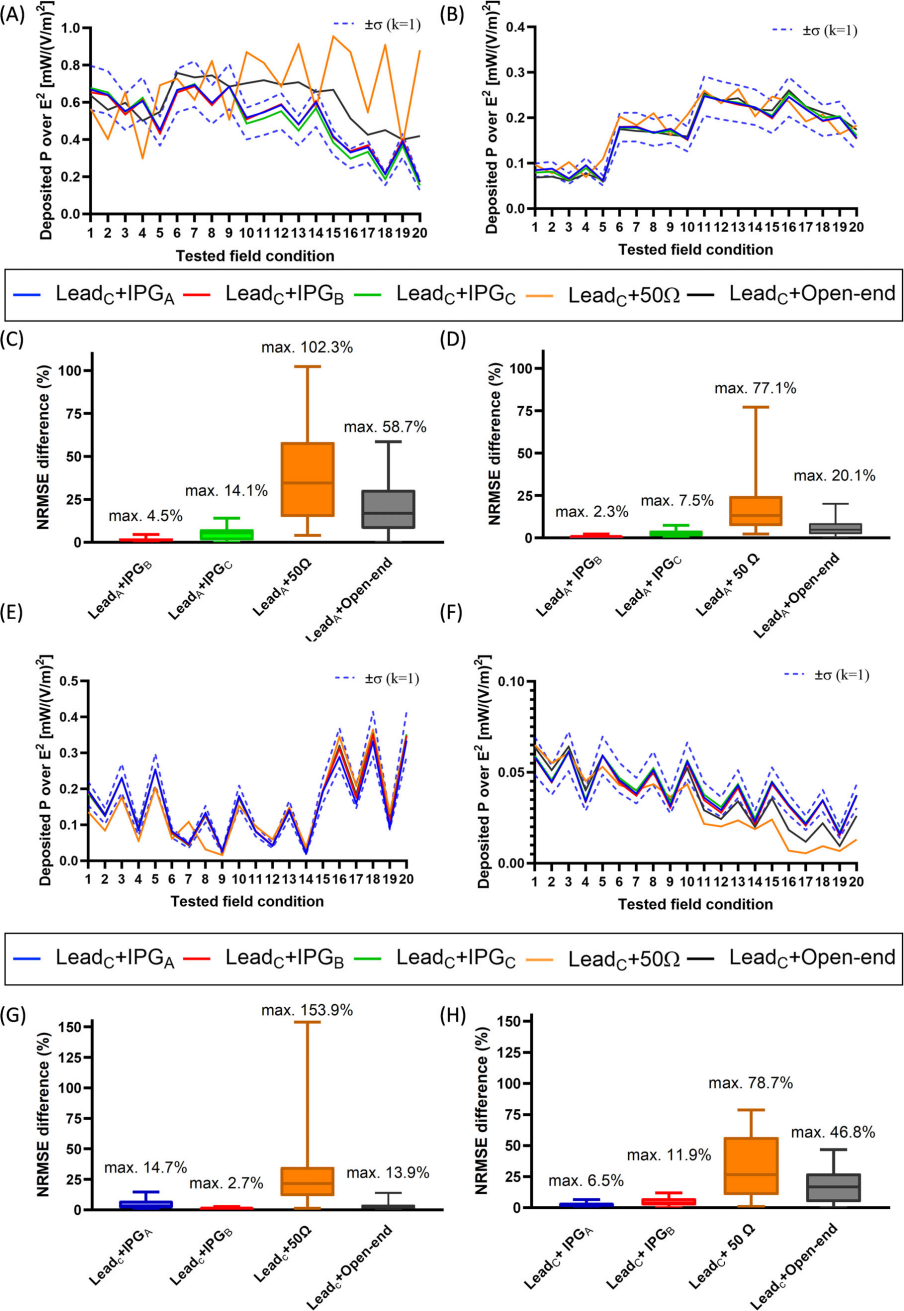
Deposited power normalized to background E‐field near the lead tip. (A, B) Normalized deposited power comparison among four conditions with confidence interval (±*σ*: 0.70 dB) for Lead A at 64 and 128 MHz, respectively; (C, D) Normalized root‐mean‐square percent error (NRMSE) difference between System A versus Lead_A_ under four conditions (IPG_B_, IPG_C_, 50 Ω, open‐ended) at 64 and 128 MHz, respectively; (E, F) Normalized deposited power comparison among four conditions with confidence interval (±*σ*: 0.70 dB) for Lead C at 64 and 128 MHz, respectively; (G, H) NRMSE difference between System C versus Lead_C_ in four conditions (IPG_A_, IPG_B_, 50 Ω, open‐ended) at 64 and 128 MHz, respectively.

RF‐induced power responses at the pacing lead electrode were assessed against 20 transmit field conditions while testing the pacing lead at the same location inside MITS‐TT by replacing the original IPG_A_ with a third‐party IPG (IPG_B_ or IPG_C_) or 50 Ω termination, and open‐ended conditions. Figure 3 shows the measured power per background electric field square (mW/(V/m)^2^) near the lead electrode, which was compared for 20 different test field conditions at 64 and 128 MHz. The total measurement uncertainty boundary (*k* = 1, *σ*: 17.6%) was plotted relative to System_A_ and System_C_, while the lead connected to the third‐party IPGs remained within the one‐sigma measurement uncertainty boundary. At 64 MHz, a maximum difference of 14.1% in measured power was observed across 20 test field conditions when the original IPG_A_ was replaced with the third‐party IPG_C_, indicating that the difference fell within the one‐sigma measurement uncertainty boundary. Compared to 102.3% and 58.7% for the pacing lead connected with 50 Ω and open‐end conditions, respectively. At 128 MHz, a maximum difference of 7.5% was found when the original IPG_A_ was replaced with the IPG_C_. This compares to the maximum differences of 77.1% and 20.1% for the pacing lead connected with 50 Ω and open‐ended conditions at 128 MHz, respectively.

RF‐induced voltage responses are shown in Figure S2. A larger change was found when System_A_ was compared with the Lead_A_ connected to IPG_C_, and 50 Ω, and remained open‐ended, where the maximum difference between System_A_ and Lead_A_‐IPG_B_ or Lead_A_‐IPG_C_ was 79.77% and 115.28% and 154.98% and 374.25% at 64 and 128 MHz, respectively. These discrepancies became larger when Lead_A_ was connected with 50 Ω, and remained open‐ended. The measured voltage was 17.3 times larger on average when Lead_A_ connected with 50 Ω and 65.5 times larger when Lead_A_ remained open‐ended under 20 incident E‐field conditions at 64 MHz. At 128 MHz, the voltages were 32.8 times and 99.9 times larger, respectively.

### 3.3 IPG Impedance Measurement

The measured impedances at 64/128 MHz are shown in Table 1, where the maximum difference between IPG_A_ and IPG_B_ or IPG_C_ was 1.64 and 4.97 Ω in magnitude at 64 and 128 MHz, respectively. The influence of IPG impedance changes (*Z*_IPG_) on the lead‐IPG system impedance (|*Z*_IPG_ + *Z*_lead_|) was maximum 1.50% at 64 MHz and 5.76% at 128 MHz. Finally, measurement uncertainties of the TF model [12] and the MITS‐TT power deposition are shown in Table 2, and RF‐induced voltage measurements in Table S2.

**TABLE 1:** Measured lead and IPG impedance (IPG_Pin_ − IPG_Can_) at 64 and 128 MHz.

64 MHz		128 MHz	
Model	Complex [Ω]	|*Z*| [Ω (∠°)]	Complex [Ω]	|*Z*| [Ω (∠°)]
Lead_A_	102.75 − j51.73	115.03 ± 0.05 (∠−26.72)	84.20−j37.05	91.99 ± 0.03 (∠−23.75)
Lead_C_	86.10 − j61.90	106.04 ± 0.05 (∠−35.71)	61.65 − j41.31	74.20 ± 0.03 (∠−33.82)
IPG_A_	2.44 + j2.30	3.36 ± 0.44 (∠43.37)	2.56 + j6.64	7.12 ± 0.53 (∠68.95)
IPG_B_	1.20 + j3.27	4.43 ± 0.24 (∠69.92)	2.88 + j7.33	7.84 ± 0.32 (∠68.55)
IPG_C_	0.57 + j1.81	2.05 ± 0.03 (∠62.34)	0.95 + j5.93	6.01 ± 0.15 (∠83.90)

**TABLE 2:** Uncertainty of the transfer function model measurement (piX) and the incident field exposure due to the tabletop *E*‐field generator (phantom‐related uncertainties were excluded as all tests were conducted under the same phantom conditions for comparison).

Source of uncertainty	Standard uncertainty (dB)
Analyzer drift (20 min)	0.01
Cable movement	0.08
TDS E‐field sensor linearity	0.25
piX Excitor positioning (*x*, *y*; 1 mm)	0.05
piX Excitor positioning (*z*; 5–20 mm)	0.25
Reflected power	0.06
Device definition (5 mm)	0.05
Post‐processing (DoF > 5)	0.07
**Combined standard uncertainty of TF model (*k* = 1)**	0.38
Tabletop test field exposure deviation	0.15
Estimating −30 dB power deposition using “*Touchless*” tool [10]	0.58
Readout electronics (SAR acquisition system) [12]	0.37
**Combined standard uncertainty of TFD experiment (*k* = 1)**	0.70

## 4 Discussion

This study aimed to develop a less burdensome test method to provide evidence of IPG impedance equivalency, which may enable leveraging originally tested MR‐Conditional labeling conditions for RF‐induced heating of mixed‐manufacturer CIED systems. This could reduce the risk of patients being injured during MRI scans, exposed to off‐label MRI scans, or being refused a necessary scan [7]. As reported by Meyers, Prutchi, and Shehada [15], the range of IPG impedances may differ depending on the vendor's choice of circuit design (measured magnitude impedance reported between 1 and 6 Ω at 64 MHz among six IPGs) similar to the pacing leads [16] (88–266 Ω at 64 MHz; unlike IPG impedance, lead characteristics are susceptible to humidity; therefore, leads should be carefully preconditioned by presoaking them in conductive liquid before testing [16, 17]). The two IPG models used in our study showed measured magnitude impedance between 2–3 Ω at 64 MHz and 6–8 Ω at 128 MHz, while the two pacing lead impedances were measured between 106–115 Ω at 64 MHz and 74–92 Ω at 128 MHz. The effects of IPG impedance on the resulting RF‐induced heating and the lead TF model used in the original lead‐IPG system should be verified when a different IPG model is introduced and connected to the lead in patients. According to Liu et al., the impedance characteristics of the IPG play a critical role in determining RF‐induced heating at the electrode tip by modifying current flow patterns throughout the lead conductor and affecting impedance matching conditions at both ends, as explained by the transmission line model [18, 19]. In this model, the lead tip current depends on the relationship between the tip impedance and the input impedance seen by the lead looking toward the IPG. It has been shown that IPG impedance can dramatically change the lead tip temperature rise by altering the standing wave pattern of RF currents along the lead [20, 21, 22, 23, 24]. However, small variations in IPG impedance among tested IPG brands may not significantly alter the standing wave pattern of RF‐induced currents, thus the RF‐induced heating profile of the lead‐IPG system in clinical MRI scanners (64 and 128 MHz) could remain within the measurement uncertainty range of the original system. When the difference in measured IPG input impedance was relatively small (< 2.5 Ω at 64 MHz, < 2.0 Ω at 128 MHz) compared to the lead‐IPG system impedance (> 108.1 Ω at 64 MHz, > 80.2 Ω at 128 MHz), the effect on RF‐induced heating was small compared to experimental uncertainty, in contrast to conditions such as 50‐Ω termination or open‐ended configurations. The maximum differences in lead‐IPG system impedances due to the third‐party IPG replacements were 2.2% at 64 MHz, and 2.3% at 128 MHz while the RF‐induced heating measurement uncertainty of 17.6% (±*σ*, *k* = 1). Programming CIEDs in “MR mode” prior to MR scanning is mandatory where a physician can change IPG to manufacturer‐defined settings using a telemetry wand and a programmer. Some modern IPG technologies allow automated MRI mode programming when an MR environment is present and return to the patient‐specific therapeutic parameters under normal environment [25]. We have not found significant differences in RF‐induced heating response (< 2.7%) and magnitude of TF models (*r* ≥ 0.99) between “MR mode” on and off; however, it is important to test the device for proper operation as per intended use [26] (Figure S3).

Our results are consistent with the previous results by Fall et al. [27], who reported that estimated RF‐induced heating did not change significantly when the pulse generators were switched among three models (two pacemakers and one CRT‐D manufactured by the same vendor). The contribution of the IPG impedances to the overall system impedance differed by less than 5% at 1.5 T MRI. In Fall's study, the RF‐induced heating was not significantly changed as the effects of the tested IPG impedance (2–3 Ω in magnitude, ≤ 4% impedance contribution in the lead‐IPG system) were not large enough to alter the TF model and lead tip heating generated by the incident E‐field at 64 MHz, compared with that of 50 and 150 Ω IPG impedances (> 25% and 50% impedance contribution in the lead‐resistor system). While Fall et al. established the theoretical foundation that small IPG impedance variations within a single manufacturer do not significantly affect RF‐induced heating using the same vendor IPG models at 1.5 T, our contribution addresses the critical clinical gap where patients with mixed‐brand CIEDs face potential MRI access restrictions, providing a less burdensome test method to ensure patient safety that tackles the real‐world scenario where patients undergo IPG replacement with devices from different manufacturers tested at both 1.5 and 3 T frequencies. Additionally, we designed a simple comparative study of the TF model by altering the termination resistance and feedthrough capacitances [28, 29] between the feedthrough wire (series *Z* = 2.4 + j10.4 Ω at 64 MHz) and IPG enclosure using an explanted mock IPG (Figure S4). This simple setup compared with the baseline IPG connection conditions, showed that as the difference in termination resistance increases, the TF model shape in magnitude changes more, which is consistent with the findings of Fall et al., and our own observations. These results support that the IPG impedance equivalence in RF‐induced heating may be demonstrated through similarity in TF models, RF‐induced power responses under different incident E‐fields, and IPG impedance comparison without a full evaluation of the Tier 3 method in ISO/TS 10974. We further examined the effects of feedthrough filter capacitors, which were found to have only a minor influence on the lead‐IPG system impedance and RF‐induced heating.

Fukunaga et al. [30] reported similar RF‐induced heating among different‐brand IPGs and pacing leads—consistent with some of our findings—but their study evaluated only a single incident E‐field exposure scenario, representing too small a sample size to draw definitive conclusions. Moreover, Fukunaga's methodology lacks electrical characterization of MR‐Conditional use for mixed‐manufacturer systems, and it remains unclear whether their approach is applicable across various exposure scenarios, such as different incident E‐fields at different landmarks, lead trajectories and patient populations, as specified for the Tier 3 method in ISO/TS 10974:2018. As we demonstrated, pacing leads with a 50‐Ω termination could also fall within the measurement uncertainty range in certain exposure scenarios, whereas others did not (Figure 3). Thus, it is beneficial to provide evidence for IPG equivalency in RF‐induced heating beyond testing a single incident E‐field condition. Our study mechanistically evaluated three critical electrical characteristics—including two different termination conditions—to provide evidence supporting IPG equivalency in RF‐induced heating based on the Tier 3 method defined in ISO/TS 10974.

This study evaluated a least‐burdensome approach to demonstrate that RF‐induced heating, as assessed in the original lead and IPG system, may remain unchanged when switching to a third‐party or different IPG model. This method does not require a complete understanding of the internal IPG circuit design, which is especially useful when assessing the compatibility of a third‐party IPG with a lead for MR Conditional labeling. Furthermore, this method does not involve repeating the extensive Tier 3 test procedures defined in ISO/TS 10974 [5], such as in vitro TF model validation and in vivo analysis across various transmit coil sizes, patient populations, and landmarks. This approach is especially beneficial when MR‐Conditional labeling for RF‐induced heating is applied to a lead that was previously tested with its original IPG but is now paired with a new or third‐party IPG. The proposed study specifically examined whether the deposited power remained within measurement uncertainties across various incident E‐fields, without extending the evaluation to actual tissue heating in clinical scenarios or to the biological impact of deposited power. This method can be particularly useful when lead‐IPG systems have already been fully assessed for patient safety with respect to in vivo temperature rise under Normal Operating Mode MRI scanners at 64 and 128 MHz. However, it does not account for the potential impact of deposited power on tissue.

On the other hand, the RF‐induced voltage or current flow toward the IPG was not equivalent even with relatively small impedance differences (Figures S1 and S2). Unlike RF‐induced heating, we were unable to find evidence supporting IPG equivalency in terms of RF‐induced voltage, as the voltage responses measured at the IPG did not stay within measurement uncertainty boundaries when the original IPG was replaced with third‐party models. As shown by Liu et al. using the transmission line model [18, 19], the RF‐induced voltage depends on the complex impedance relationship expressed as |*Z*_IPG_/(*Z*_IPG_ + *Z*_lead_)|, where *Z*_lead_ is the total input impedance of the lead. This relationship makes the induced voltage at the IPG highly sensitive to changes in IPG impedance. The IPG impedance can vary depending on the manufacturer's design of the feedthrough wires connected to the IPG circuitry [15] and the electromagnetic interference (EMI) filter capacitors used to protect the IPG circuitry [28, 29]. Since the RF‐induced voltage is a characteristic seen at the IPG circuitry, even slight differences in these design parameters can noticeably alter the induced‐voltage outcome. Therefore, a more conservative but less burdensome test method than the Tier 3 approach defined in ISO/TS 10974 may be practical for evaluating RF‐induced voltage effects on IPG circuits when replacing one IPG model with another. We recently performed a systematic evaluation of the effects of capacitive terminations on the TF model of a mock lead/IPG system at 1.5 T ranging between 100 pF and 10 μF [31]. This previous study showed similar results where small variations in capacitance did not significantly alter the heating profile, whereas the voltage response was more sensitive to changes even with capacitance variation. In the absence of the original lead/IPG system, it was not possible to compare different conditions with the clinically representative third‐party IPG models, even though the study was conducted using the exhaustive full Tier 3 process as defined in ISO/TS 10974.

### 4.1 Limitations

Our study was conducted using two pacing leads and three IPGs from three different manufacturers. Other IPGs may exhibit larger impedance variations than those tested in this study or reported in previous work. In this study, clinically typical pacing lead lengths (e.g., 55 and 89 cm) were tested; these leads are longer than the half‐wavelengths at 64 MHz or 128 MHz. The wavelength‐dependent effects on leads lengths approaching resonant conditions (near the half‐wavelength) may warrant further study [32].

## 5 Conclusion

In this study, we demonstrated that simplified IPG interchangeability test methods, TF models, IPG impedance and a deposited power comparison showed evidence of IPG interchangeability between a representative original IPG and third‐party IPGs at both 64 and 128 MHz. We presented a least‐burdensome approach to demonstrate the IPG equivalency condition such that the original configuration's MR Conditional labeling may potentially address MRI safety when a different IPG model is implanted in a CIED patient. While standard methods per ISO/TS 10974 would prescribe new RF‐induced heating evaluation for a new mixed‐component system, our results suggest that in some cases, if IPG electrical equivalency on the lead heating can be demonstrated using the TF model and deposited power response at the lead tip, the MR safety profile may not change significantly compared to that of the original system configuration. Use of this approach may facilitate efficient MR safety evaluation of mixed‐manufacturer CIED systems, ultimately expanding patient access to medically necessary MRI scans.

## Funding

The authors have nothing to report.

## Supporting information


**Figure S1:** Normalized RF‐induced voltage transfer function models of a pacing lead under five conditions $\left(1=\int_{0}^{L}\mathrm{TF}\left(l\right)\mathrm{dl}\right){\left(\int_{0}^{L}\mathrm{TF}\left(l\right)\mathrm{dl}\right)}^{*}$. (A) Measured voltage transfer function model at 64 MHz (1.5 T); (B) Measured voltage transfer function model at 128 MHz (3 T).
**Figure S2:** RF‐induced voltage measured at the IPG_electrode‐pin_ at 64 MHz and 128 MHz. (A) RF‐induced voltage measurements at 20 different incident E‐field conditions at 64 MHz; (B) Zoomed view of RF‐induced voltage for a pacing lead under three conditions with one‐sigma measurement uncertainty boundary (±*σ*: 1.17 dB) plotted in dashed lines; (C) RF‐induced voltage measurements at 20 different incident E‐field conditions at 128 MHz; (D) Zoomed view of RF‐induced voltage for a pacing lead under three conditions with one sigma measurement uncertainty boundary plotted in dashed lines.
**Figure S3:** Unscaled measured transfer function (TF) model of a pacing lead when “MRI mode” On and Off at (A) 64 MHz and (B) 128 MHz. (correlation 0.99). The maximum NRMSE difference in RF‐induced heating response across 20 incident E‐field conditions between MRI mode On and Off was 2.63%.
**Figure S4:** Normalized transfer function (TF) model of a pacing lead $\left(1=\int_{0}^{L}\mathrm{TF}\left(l\right)\mathrm{dl}\right){\left(\int_{0}^{L}\mathrm{TF}\left(l\right)\mathrm{dl}\right)}^{*}$ connected to IPG A vs. a mock IPG with feedthrough wire without IPG circuitry connections replaced with (A) resistive termination and (B) shunt capacitors at 64 MHz, and (C) resistive termination and (D) shunt capacitors at 128 MHz, all grounded to the IPG enclosure.
**Table S1:** A set of test fields to generate various incident fields with relative phase differences in the range between 0° and 180° and relative amplitude differences in the range between −6 and 6 dB. This test field set is used to uniformly cover the relative phase and amplitude differences between the two transmit sources of a tabletop E‐field generator.
**Table S2:** Uncertainty of the RF‐induced voltage measurement due to the TDS RFoF1P4MED System1 and the incident field exposure due to the tabletop E‐field generator (phantom‐related uncertainties were excluded as all tests were conducted under the same phantom conditions for comparison).

## Data Availability

The data that support the findings of this study are available from the corresponding author upon reasonable request.

## References

[1] J. A. Ladapo , C. E. Spritzer , X. V. Nguyen , J. Pool , and E. Lang , “Economics of MRI Operations After Implementation of Interpersonal Skills Training,” Journal of the American College of Radiology 15, no. 12 (2018): 1775–1783, 10.1016/j.jacr.2018.01.017. 29530323 PMC6129443

[2] L. Al‐Dayeh , M. Rahman , and R. Venook , “Practical Aspects of MR Imaging Safety Test Methods for MR Conditional Active Implantable Medical Devices,” Magnetic Resonance Imaging Clinics of North America 28, no. 4 (2020): 559–571, 10.1016/j.mric.2020.07.008. 33040996

[3] J. G. Delfino , D. M. Krainak , S. A. Flesher , and D. L. Miller , “MRI‐Related FDA Adverse Event Reports: A 10‐Yr Review,” Medical Physics 46, no. 12 (2019): 5562–5571, 10.1002/mp.13768. 31419320

[4] J. M. Henderson , J. Tkach , M. Phillips , K. Baker , F. G. Shellock , and A. R. Rezai , “Permanent Neurological Deficit Related to Magnetic Resonance Imaging in a Patient With Implanted Deep Brain Stimulation Electrodes for Parkinson's Disease: Case Report,” Neurosurgery 57, no. 5 (2005): E1063, 10.1227/01.NEU.0000180810.16964.3E. 16284543

[5] ISO/TS 10974:2018 , “Assessment of the Safety of Magnetic Resonance Imaging for Patients With an Active Implantable Medical Device,” (2018), https://www.iso.org/standard/65055.html.

[6] C. A. König , F. Tinhofer , T. Puntus , et al., “Is Diversity Harmful?—Mixed‐Brand Cardiac Implantable Electronic Devices Undergoing Magnetic Resonance Imaging,” Wiener Klinische Wochenschrift 134, no. 7–8 (2022): 286–293, 10.1007/s00508-021-01924-w. 34402991 PMC9023390

[7] R. J. Russo , L. Gakenheimer‐Smith , U. M. Birgersdotter‐Green , et al., “HRS Call‐to‐Action: Improved MRI Access for Patients With Cardiovascular Implantable Electronic Devices,” Heart Rhythm 22 (2025): e821–e836, 10.1016/j.hrthm.2025.04.028. 40294730

[8] E. Zastrow , M. Capstick , and N. Kuster , “Experimental Systems for RF‐Heating Characterization of Medical Implants During MRI,” in Proceedings 24. Annual Meeting International Society for Magnetic Resonance in Medicine. 24 (ISMRM, 2016), 0912.

[9] A. Yao , E. Zastrow , E. Neufeld , M. Cabanes‐Sempere , T. Samaras , and N. Kuster , “Novel Test Field Diversity Method for Demonstrating Magnetic Resonance Imaging Safety of Active Implantable Medical Devices,” Physics in Medicine and Biology 65, no. 7 (2020): 075004, 10.1088/1361-6560/ab7507. 32045896

[10] A. Yao , E. Zastrow , and N. Kuster , “Data‐Driven Experimental Evaluation Method for the Safety Assessment of Implants With Respect to RF‐Induced Heating During MRI,” Radio Science 53, no. 6 (2018): 700–709, 10.1029/2017RS006433.

[11] H. Jeong , J. W. Guag , and A. Kumar , “RF‐Induced Heating Estimation of a Stent in a 3T MRI Using Transfer Function Approach With a Tabletop E‐Field Generator,” IEEE Access 12 (2024): 191945, 10.1109/ACCESS.2024.3518974. 41041652 PMC12486246

[12] J. Córcoles , A. Yao , and N. Kuster , “Experimental and Numerical Optimization Modelling to Reduce Radiofrequency‐Induced Risks of Magnetic Resonance Examinations on Leaded Implants,” Applied Mathematical Modelling 96 (2021): 177–188, 10.1016/j.apm.2021.02.036.

[13] ZMT Validation Hardware , Application Note: Assessing Terminal Voltages Under MRI Exposure With RFoF1P4MED (ZMT Validation Hardware, 2021).

[14] ISO/IEC Guide 98‐3:2008 , Uncertainty of Measurement—Part 3 Guide to the Expression of Uncertainty in Measurement (ISO/IEC Guide, Edition 1, 2008), https://www.iso.org/standard/50461.html.

[15] J. Meyers , D. Prutchi , and R. Shehada , “Input Impedance Comparison of MR‐Conditional Cardiac Implantable Pulse Generators at the 1.5T MR Frequency of 63.87 MHz,” in Proceedings 29. Annual Meeting International Society for Magnetic Resonance in Medicine, vol. 29 (International Society for Magnetic Resonance in Medicine, 2021), 2311, https://archive.ismrm.org/2021/2311.html.

[16] D. Prutchi , J. Meyers , and R. Shehada , “Importance of Pacemaker Lead Preconditioning for MR Safety In‐Vitro Studies,” in Proceedings 29. Annual Meeting International Society for Magnetic Resonance in Medicine, vol. 29 (International Society for Magnetic Resonance in Medicine, 2021), 2282, https://archive.ismrm.org/2021/2282.html.

[17] ANSI/AAMI PC76:2021 , “Active Implantable Medical Devices–Requirements and Test Protocols for Safety of Patients With Pacemakers and ICDs Exposed to Magnetic Resonance Imaging,” (2021), https://webstore.ansi.org/standards/aami/ansiaamipc762021?srsltid=AfmBOorFzQBIEMXiWtBPlb0tyVCEQhKYu_XLlXnAUCb5GpiVCeysTDWo.

[18] J. Liu , J. Zheng , Q. Wang , W. Kainz , and J. Chen , “A Transmission Line Model for the Evaluation of MRI RF‐Induced Fields on Active Implantable Medical Devices,” IEEE Transactions on Microwave Theory and Techniques 66, no. 9 (2018): 4271–4281, 10.1109/TMTT.2018.2851975.

[19] J. Liu , D. R. Jackson , Q. Wang , W. Kainz , and J. Chen , “On the Relationship Between Impedances of Active Implantable Medical Devices and Device Safety Under MRI RF Emission,” IEEE Trans Electromagn Compat. Published Online PP (99) (2019): 1–9, 10.1109/TEMC.2019.2938908.

[20] V. Acikel and E. Atalar , “Modeling of Radio‐Frequency Induced Currents on Lead Wires During MR Imaging Using a Modified Transmission Line Method,” Medical Physics 38, no. 12 (2011): 6623–6632, 10.1118/1.3662865. 22149844

[21] V. Acikel , A. Uslubas , and E. Atalar , “Modeling of Electrodes and Implantable Pulse Generator Cases for the Analysis of Implant Tip Heating Under MR Imaging,” Medical Physics 42, no. 7 (2015): 3922–3931, 10.1118/1.4921019. 26133593

[22] V. Acikel , B. Silemek , and E. Atalar , “Wireless Control of Induced Radiofrequency Currents in Active Implantable Medical Devices During MRI,” Magnetic Resonance in Medicine 83, no. 6 (2020): 2370–2381, 10.1002/mrm.28089. 31763729

[23] A. C. Özen , T. Lottner , and M. Bock , “Safety of Active Catheters in MRI: Termination Impedance Versus RF‐Induced Heating,” Magnetic Resonance in Medicine 81, no. 2 (2019): 1412–1423, 10.1002/mrm.27481. 30346056

[24] A. C. Özen , B. Silemek , T. Lottner , E. Atalar , and M. Bock , “MR Safety Watchdog for Active Catheters: Wireless Impedance Control With Real‐Time Feedback,” Magnetic Resonance in Medicine 84, no. 2 (2020): 1048–1060, 10.1002/mrm.28153. 31961965

[25] S. Mullane , K. Michaelis , C. Henrikson , et al., “Utilization and Programming of an Automatic MRI Recognition Feature for Cardiac Rhythm Management Devices,” Heart Rhythm O2 2, no. 2 (2021): 132–137, 10.1016/j.hroo.2021.03.002. 34113915 PMC8183951

[26] U.S. Food and Drug Administration , “Testing and Labeling Medical Devices for Safety in the Magnetic Resonance (MR) Environment, Guidance for Industry and Food and Drug Administration Staff,” (2023), https://www.fda.gov/media/74201/download.

[27] A. K. Fall , A. R. Guraliuc , N. Monfret , and R. Setzu , “MRI Conditional Safety of Mixed Systems: Study of the Impact of Active Implant Medical Device Impedance on the Radiofrequency‐Induced Lead Heating,” Magnetic Resonance in Medicine 89, no. 6 (2023): 2332–2346, 10.1002/mrm.29583. 36655698

[28] R. A. Stevenson , “Feedthrough EMI Filter With Ground Isolation for Cardiac Pacemakers and Implantable Cardioverter Defibrillators,” in Proceedings of the 20th Annual International Conference of the IEEE Engineering in Medicine and Biology Society. Vol. 20 Biomedical Engineering Towards the Year 2000 and Beyond (Cat. No. 98CH36286), vol. 6 (IEEE, 1998), 3319–3323, 10.1109/IEMBS.1998.746210.

[29] R. A. Stevenson , “Design and Application of Broadband Ceramic Feedthrough Capacitor EMI Filters to Cardiac Pacemakers and Implantable Defibrillators,” in Proceedings of the 19th Annual International Conference of the IEEE Engineering in Medicine and Biology Society. “Magnificent Milestones and Emerging Opportunities in Medical Engineering” (Cat. No.97CH36136), vol. 6 (IEEE, 1997), 2558–2562, 10.1109/IEMBS.1997.756852.

[30] I. Fukunaga , S. Shibukawa , S. Yatsushiro , et al., “MR‐Safety of Mixed‐Brand of Cardiac Implantable Electronic Devices: Comparison of RF Induced Heating With Approved Single‐Brand at 1.5 T and 3.0 T,” in Proceedings 31. Annual Meeting International Society for Magnetic Resonance in Medicine, vol. 31 (International Society for Magnetic Resonance in Medicine, 2023), 5433, https://archive.ismrm.org/2023/5433.html.

[31] F. Ebrahimi , M. K. Akter , Q. Wang , et al., “Numerical and Experimental Study on Mixed Use of Implantable Pulse Generator for AIMDs Under 1.5T MRI,” IEEE Transactions on Electromagnetic Compatibility PP (99) (2026): 1–9, 10.1109/TEMC.2025.3650337.

[32] C. J. Yeung , R. C. Susil , and E. Atalar , “RF Safety of Wires in Interventional MRI: Using a Safety Index,” Magnetic Resonance in Medicine 47, no. 1 (2002): 187–193, 10.1002/mrm.10037. 11754458

