# Supplementary material for: An Interchangeability Test Method for Developing IPG Impedance Equivalency Evidence for Cardiac Implantable Electronic Device Under MRI
Source: Magn Reson Med. 2026 Apr 26;96(2):883–91. doi: 10.1002/mrm.70389 (PMC13269235; doi:10.1002/mrm.70389)
Supplement: Supplementary file 1 — Figure S1: Normalized RF‐induced voltage transfer function models of a pacing lead under five conditions 1=∫0LTF(l)dl∫0LTF(l)dl*. (A) Measured voltage transfer function model at 64 MHz (1.5 T); (B) Measured voltage transfer function model at 128 MHz (3 T). Figure S2: RF‐induced voltage measured at the IPGelectrode‐pin at 64 MHz and 128 MHz. (A) RF‐induced voltage measurements at 20 different incident E‐field conditions at 64 MHz; (B) Zoomed view of RF‐induced voltage for a pacing lead under three conditions with one‐sigma measurement uncertainty boundary (±σ: 1.17 dB) plotted in dashed lines; (C) RF‐induced voltage measurements at 20 different incident E‐field conditions at 128 MHz; (D) Zoomed view of RF‐induced voltage for a pacing lead under three conditions with one sigma measurement uncertainty boundary plotted in dashed lines. Figure S3: Unscaled measured transfer function (TF) model of a pacing lead when “MRI mode” On and Off at (A) 64 MHz and (B) 128 MHz. (correlation 0.99). The maximum NRMSE difference in RF‐induced heating response across 20 incident E‐field conditions between MRI mode On and Off was 2.63%. Figure S4: Normalized transfer function (TF) model of a pacing lead 1=∫0LTF(l)dl∫0LTF(l)dl* connected to IPG A vs. a mock IPG with feedthrough wire without IPG circuitry connections replaced with (A) resistive termination and (B) shunt capacitors at 64 MHz, and (C) resistive termination and (D) shunt capacitors at 128 MHz, all grounded to the IPG enclosure. Table S1: A set of test fields to generate various incident fields with relative phase differences in the range between 0° and 180° and relative amplitude differences in the range between −6 and 6 dB. This test field set is used to uniformly cover the relative phase and amplitude differences between the two transmit sources of a tabletop E‐field generator. Table S2: Uncertainty of the RF‐induced voltage measurement due to the TDS RFoF1P4MED System1 and the incident field exposure due to the tabletop E [file MRM-96-883-s001.docx]

Supporting Information

**An interchangeability test method for developing IPG impedance equivalency evidence for Cardiac Implantable Electronic Device (CIED) under MRI**

Hongbae Jeong^1*^, Andrew Smith^2^, Ananda Kumar^1^

^1^Office of Science and Engineering Laboratories, Center for Devices and Radiological Health, U. S. Food and Drug Administration, Silver Spring, MD 20993 USA

^2^Office of Cardiovascular Devices, Center for Devices and Radiological Health, U. S. Food and Drug Administration, Silver Spring, MD 20993 USA

*Corresponding author: Hongbae Jeong (e-mail: hongbae.jeong@fda.hhs.gov)

1. Voltage transfer function model and RF-induced voltage response measurement results using a tabletop E-field generator at 64 MHz and 128 MHz

The voltage response transfer function model of the device was further measured using a piecewise excitation (piX; ZMT, Zurich MedTech AG, Switzerland) system, which characterizes current flow toward the IPG circuit (Supporting Information Figure S1). The time-domain sensor electro-optical voltage probe system (TDS RFoF1P4MED system; Schmid & Partners Engineering AG; SPEAG, Switzerland) equipped with four rubber O-rings was positioned inside a 3D-printed cylindrical sleeve which provides a water-tight connection of the probe to the standard 851 two-pin connector (Preci-Dip, USA). Two pins on the 851 connector were then connected to the lead electrode pin on the IPG socket and the IPG housing with copper tape and sealed using an adhesive sealant (GOOP All Purpose Adhesive; Electric Products, Inc., USA). A pacing lead and IPG from the same vendor were positioned on a straight holder as done for the RF-induced heating transfer function (TF) model measurement and submerged in conductive tissue simulation liquid (TSL; σ: 0.47 S/m, ε_r_: 78; TLe78c0.47, ZMT) while the piX excitor coil was swept along the pacing lead. The measured voltage response was processed using a TDS remote unit (1RU1TDS, SPEAG) and logged directly using the piX system. The voltage TF model measurement was repeated under the same exposure conditions while maintaining the same lead position. The original IPG_A_ was then replaced with a third-party IPG (IPG_B_), a 50 Ω resistor, followed by a lead open-circuit condition at the IPG.

|  |
| --- |
| Supporting Information Figure S1. Normalized RF-induced voltage transfer function models of a pacing lead under five conditions $\boldsymbol{(1=}\int_{\boldsymbol{0}}^{\boldsymbol{L}} \boldsymbol{TF}\left( \boldsymbol{l} \right)\boldsymbol{dl)}{\boldsymbol{(}\int_{\boldsymbol{0}}^{\boldsymbol{L}} \boldsymbol{TF}\left( \boldsymbol{l} \right)\boldsymbol{dl}\boldsymbol{)}}^{\boldsymbol{*}}$). (A) Measured voltage transfer function model at 64 MHz (1.5T); (B) Measured voltage transfer function model at 128 MHz (3T). |

Furthermore, the RF-induced voltage responses were measured by placing the CIED system submerged in conductive TSL and exposing it to twenty different incident E-fields from a tabletop E-field generator (MITS-TT, ZMT). The signals measured using the RFoF1P4MED system in dBm were assessed using a Vector Network Analyzer (P5004A; Keysight Technologies, USA) in receive mode at 64 and 128 MHz, and then converted into voltages (Supporting Information Figure S2).

|  |
| --- |
| Supporting Information Figure S2. RF-induced voltage measured at the IPG_electrode-pin_ at 64 MHz and 128 MHz. (A) RF-induced voltage measurements at twenty different incident E-field conditions at 64 MHz; (B) Zoomed view of RF-induced voltage for a pacing lead under three conditions with one-sigma measurement uncertainty boundary (±σ: 1.17dB) plotted in dashed lines; (C) RF-induced voltage measurements at twenty different incident E-field conditions at 128 MHz; (D) Zoomed view of RF-induced voltage for a pacing lead under three conditions with one sigma measurement uncertainty boundary plotted in dashed lines.    Supporting Information Figure S3. Unscaled measured transfer function (TF) model of a pacing lead when ‘MRI mode’ On and Off at (A) 64 MHz and (B) 128 MHz. (correlation ≥0.99). The maximum NRMSE difference in RF-induced heating response across 20 incident E-field conditions between MRI mode On and Off was 2.63%.    Supporting Information Figure S4. Normalized transfer function (TF) model of a pacing lead $\boldsymbol{(1=}\int_{\boldsymbol{0}}^{\boldsymbol{L}} \boldsymbol{TF}\left( \boldsymbol{l} \right)\boldsymbol{dl)}{\boldsymbol{(}\int_{\boldsymbol{0}}^{\boldsymbol{L}} \boldsymbol{TF}\left( \boldsymbol{l} \right)\boldsymbol{dl}\boldsymbol{)}}^{\boldsymbol{*}}$) connected to IPG A vs. a mock IPG with feedthrough wire without IPG circuitry connections replaced with (A) resistive termination and (B) shunt capacitors at 64 MHz, and (C) resistive termination and (D) shunt capacitors at 128 MHz, all grounded to the IPG enclosure. |

**Supporting Information** **Table S1.** A set of test fields to generate various incident fields with relative phase differences in the range between 0° and 180° and relative amplitude differences in the range between -6 dB and 6 dB. This test field set is used to uniformly cover the relative phase and amplitude differences between the two transmit sources of a tabletop E-field generator.

| **Test Field Index** | **Phase difference (°)** | **Relative amplitude difference (dB)** | **Test Field Index** | **Phase difference (°)** | **Relative amplitude difference (dB)** |
| --- | --- | --- | --- | --- | --- |
| **1** | 0 | 0 | **11** | 120 | 0 |
| **2** | 0 | 3 | **12** | 120 | 3 |
| **3** | 0 | -3 | **13** | 120 | -3 |
| **4** | 0 | 6 | **14** | 120 | 6 |
| **5** | 0 | -6 | **15** | 120 | -6 |
| **6** | 60 | 0 | **16** | 180 | 0 |
| **7** | 60 | 3 | **17** | 180 | 3 |
| **8** | 60 | -3 | **18** | 180 | -3 |
| **9** | 60 | 6 | **19** | 180 | 6 |
| **10** | 60 | -6 | **20** | 180 | -6 |

**Supporting Information Table S2.** Uncertainty of the RF-induced voltage measurement due to the TDS RFoF1P4MED System^1^ and the incident field exposure due to the tabletop E-field generator. (Phantom-related uncertainties were excluded as all tests were conducted under the same phantom conditions for comparison)

| Source of uncertainty | Std. Uncertainty (dB) |
| --- | --- |
| **Calibration** | |
| RFoF4MEDCU transmission calibration | 0.87 |
| Mismatch RFoF4MEDCU (<-20 dB), VNA (<-16dB) | 0.10 |
| Mismatch remote unit (<-10 dB), VNA (<-16dB) | 0.30 |
| Differential Connector | 0.30 |
| Repeatability | 0.60 |
| **RFoFSystem** | |
| Linearity | 0.50 |
| Fiber optic connector repeatability | 0.11 |
| Fiber optic repeatability | 0.50 |
| **Signal Measurement** | |
| Mismatch remote unit (<-10dB), FSP30 (<-14 dB) | 0.38 |
| Signal analyzer total measurement uncertainty | 0.50 |
| **Combined Standard Uncertainty of Voltage measurement (k=1)** | 1.49 |
| Source of Uncertainty | Std. Uncertainty (dB) |
| Tabletop test field exposure deviation | 0.15 |
| Readout electronics (Voltage measurement system, k=1)^1^ | 1.16 |
| **Combined Standard Uncertainty of TFD experiment (k=1)** | 1.17 |

Disclaimer

The mention of commercial products, their sources, or their use in connection with material reported herein is not to be construed as an actual or implied endorsement of such products by the U.S. Department of Health and Human Services.

Supporting Information References

1. ZMT Validation Hardware. *Application Note: Assessing Terminal Voltages under MRI Exposure with RFoF1P4MED*.; 2021.
